# The effectiveness of acupuncture and moxibustion for treating tenosynovitis: A systematic review and meta-analysis protocol

**DOI:** 10.1097/MD.0000000000022372

**Published:** 2020-12-04

**Authors:** Shouqiang Huang, Qian Fan, Jun Xiong, Kai Liao, Fanghui Hua, Jie Xiang, Changsheng Li, Hangyu Jin

**Affiliations:** a Jiangxi University of Traditional Chinese Medicine, Nanchang; b Changshu Hospital of Traditional Chinese Medicine, Changshu; c The Affiliated Hospital of Jiangxi University of Traditional Chinese Medicine, Nanchang, China.

**Keywords:** De Quervain, tenosynovitis, acupuncture and moxibustion, protocol, systematic review

## Abstract

**Background:**

De Quervain's tenosynovitis is an overuse disease that involves a thickening of the abductor pollicis longus and extensor pollicis brevis tendons. Evidence shows that acupuncture and moxibustion (AM) could remarkably relieve the pain of De Quervain's tenosynovitis patients. The aim of this protocol is to determine the efficacy and safety of AM for treating De Quervain's tenosynovitis.

**Methods:**

Several online databases including PubMed, Cochrane Library, Embase, Chinese Biomedical Literatures Database, China National Knowledge Infrastructure, WangFang Database, Chinese Scientific Journal Database will be searched from their establishment to July 1, 2020. We will include all randomized controlled trials using AM as the method for treating De Quervain's tenosynovitis, regardless of blinding or publication types. The selection of studies, data extraction and the assessment of the studies quality will be conducted by 2 reviewers separately. When there is sufficient available data for meta-analysis, we will use the RevMan V.5.3 statistical software for data synthesis. The total effective rate, range of motion of wrist ulnar deviation will be the primary outcomes, and the secondary outcomes contain Visual Analog Scale, Coney Wrist Score and side effects. We will express the result by using Risk ratio for dichotomous data and mean differences with a 95% confidence interval for continuous data.

**Results:**

The results of this study be presented in corresponding journal or conferences.

**Conclusions:**

This study is designed to provide sufficient evidence to assess the exact effectiveness of AM on De Quervain's tenosynovitis.

**PROSPERO registration number:**

CRD42020158764.

## 1 Introduction

Tenosynovitis is a painful condition that often involves tendons and their supportive soft-tissue structures of the hand, wrist and forearm. According to clinical manifestations, tenosynovitis can be divided into following 5 types: stenosing tenosynovitis, acute suppurative tenosynovitis, acute fibrous tenosynovitis, acute serous tenosynovitis, tuberculous tenosynovitis, of which the stenosing tenosynovitis is the most common type. De Quervain's tenosynovitis, as a commonplace stenosing tenosynovitis, will be the emphasis of this protocol.

De Quervain's tenosynovitis is entrapment tendinitis of the tendons within the first dorsal compartment that involves a thickening of the extensor retinaculum.^[1]^ Its clinical symptoms may manifest oedema, the pain of the radial styloid process, and weakened grip strength,^[2]^ which is mainly due to the thickening of extensor retinaculum and compression of abductor pollicis longus (APL) and extensor pollicis brevis (EPB) tendons, then results in the limited sliding of APL and EPB tendons in the narrow chamber.^[3–5]^ According to the clinical manifestations, De Quervain's tenosynovitis should belong to the category of tendon injury and arthralgia syndrome in traditional Chinese medicine, caused by the wind-cold-wetness evil and overwork. De Quervain's tenosynovitis usually occurs in people engaged in repetitive or forceful manual work and pregnancy,^[6]^ and the symptoms tend to increase when the thumb moves, such as gripping a golf club, lifting a child, wringing of cloth. On clinical examination, it may reveal swelling and tenderness in the region of the first dorsal compartment, and Finklestein's test turned positive.^[7]^

The proportion of women with De Quervain's tenosynovitis is typically more in women than that of men. A study revealed that the prevalence of De Quervain's tenosynovitis is estimated at 0.5% in males and 1.3% in female.^[8]^ In recent years, owing to modern living conditions such as excessive use of computers and increased use of mobile phones, the incidence of De Quervain's tenosynovitis has gradually increased.^[9]^ It is reported that De Quervain's tenosynovitis costs the US economy between $13 billion and $20 billion each year.^[10]^

Treatment of De Quervain's tenosynovitis varies according to the severity of the disease, which roughly can divide into conservative and surgical managements. The conservative managements, as the first line of treatment, include anti-inflammatory medication, corticosteroid injections, and occupational therapy(OT).^[7]^ Although these therapies can effectively relieve symptoms to some degree, they frequently accompany by some shortcomings. Taking corticosteroid injections as an example, that may have a potential local infection, atrophy of subcutaneous fat even tendon rupture risks.^[11]^ When conservative treatment fails, surgery to relieve pressure in the first dorsal chamber may be performed, but in essence, it is an invasive intervention involved many surgical relevant risks.^[12]^ There are more options for traditional Chinese medicine, such as acupuncture, moxibustion, fire needle, small needle knife warm needle etc. And it has the characteristics of simple operation and definite curative effect.

Acupuncture and moxibustion (AM), as a significant component of Traditional Chinese Medicine, has played a full advantage in the treatment of various diseases for thousands of years. In modern-day society, the global interest and demand for this therapy are also increasing.^[13]^ It has been the most common well-recognized complementary and alternative treatment for various musculoskeletal pain diseases.

The mechanism of action for AM treating these diseases may be due to the improvement of local blood supply, connective tissue and autonomic nerve activity, as well as regulation of anti-inflammatory actions.^[14,15]^

Evidence showed that AM is valid for treating De Quervain's tenosynovitis,^[16–18]^ which may improve the local blood circulation of the lesion, promote the repair of muscles, tendons and other soft tissues. A previous systematic review and meta-analyses published in Chinese also described the effect of AM on De Quervain's tenosynovitis, and it may have some advantages compared with traditional treatments.^[19]^

However, due to heterogeneity and methodological limitations, and the evidence supporting its efficacy is seemed not acknowledgeable from the perspective of evidence-based medicine. Additionally, although there was 1 systematic review^[19]^ reported the effectiveness of AM on De Quervain's tenosynovitis, it only included 4 randomized controlled trials (RCTs). It didn’t involve other outcomes such as visual analog scale, Cooney wrist joint rating scale and side effects. Hence, strong evidence is still needed to solve this problem, such as RCTs of rigorous trial design methods and a large, multicentre sample. Given this situation, we reassess the issue by collecting more comprehensive evidence to conduct a systematic review and meta-analysis, hoping to determine the efficacy of AM in De Quervain's tenosynovitis.

## 2 Objective

The aim of this protocol is designed to summarizing the clinical evidence of AM for treating De Quervain's tenosynovitis in hope that can be utilized by doctors, patients, policy decision-makers.

## 3 Methods and analysis

### 3.1 Study registration

This study protocol was recorded in the PROSPERO as CRD42020158764, and we could obtain from URL: http://www.crd.york.ac.uk/PROSPERO/display_record.php?ID=CRD42020158764. The written document of this protocol will be conducted following the systematic review and meta-analysis protocol (PRISMA-P) statement in the Cochrane Hand book.^[20]^ If necessary, we will describe some changes in our full review.

### 3.2 Inclusion criteria

#### 3.2.1 Type of studies

All RCTs presented in English and Chinese regarding AM for De Quervain's tenosynovitis will be included. No restriction exists on blinding or publication types.

#### 3.2.2 Type of participants

Participants refer to patients diagnosed as De Quervain's tenosynovitis, and there is no restrictions about gender, age, job, disease duration, and ethnicity. Diagnosis of De Quervain's tenosynovitis is according to Chinese diagnostic criteria for De Quervain's tenosynovitis.^[21]^

#### 3.2.3 Type of interventions

The interventions of the treatment group will comprise of participants receiving conventional acupuncture, electroacupuncture, fire needle, floating needle, moxibustion and other forms of AM treatment. Besides, some combination therapy with acupuncture or moxibustion will also be included, such as acupuncture plus western medicine, acupuncture plus massage. With on limit to the retaining time, frequency and course of treatment.

#### 3.2.4 Type of comparators

The interventions of the control group will comprise of participants receiving sham acupuncture or moxibustion, placebo, western medicine, routine care, massage and other active therapies. There are no restrictions on the frequency and duration of treatment.

#### 3.2.5 Types of outcome measures

##### 3.2.5.1 Primary outcomes

The primary outcomes mainly include the following aspects in this review

(1) Total effective rate
(2) Range of motion of wrist ulnar deviation: The measurement value refers to the angle between the remote forearm and the third metacarpal bone, and taking the head-shaped carpal bone on the back of the palm as the centre point.

##### 3.2.5.2 Secondary outcomes

The secondary outcomes of this review mainly consist of the following aspects:

(1) Side effects
(2) Visual analog scale
(3) Coney Wrist Score: Using the Cooney wrist score scale^[22]^ to assess the pain, functional status, range of motion, grip strength and other indexes of the wrist.

### 3.3 Exclusion certain for study selection

The exclusion criteria in this review are basically considered from the following items:

(1) Comparative study on the therapeutic effects of different AM
(2) Tenosynovitis caused by other factors or patients with other serious diseases
(3) The literature published repeatedly or unable to obtain the full text

### 3.4 Search strategy

#### 3.4.1 Electronic searches

Several online databases including PubMed, Cochrane Library, Embase, Chinese Biomedical Literatures Database, China National Knowledge Infrastructure, WangFang Database, Chinese Scientific Journal Database will be searched from their establishment to July 1, 2020. The literature published in Chinese or English will be included. We will choose the subject headings and free terms as the search terms. The search terms will be consist of disease term part(eg, De Quervain's tenosynovitis OR De Quervain stenosing tenosynovitis OR De Quervain disease OR Stenosing tenosynovitis), the intervention term part(eg, AM OR Acupuncture OR moxibustion OR Electroacupuncture OR Fire needle) and the study type(eg, randomized controlled trial OR controlled clinical trial OR randomized OR randomly). Table 1 presents the searching strategy of PubMed.

**Table 1:** Search Strategy (PubMed).

Order	Strategy
#1	Search “de quervain's tenosynovitis”[Mesh] OR“tenosynovitis”[Mesh] Sort by: Publication Date
#2	Search ((((de quervain's tenosynovitis[Title/Abstract]) OR de Quervain stenosing tenosynovitis [Title/Abstract]) OR de quervain disease[Title/Abstract]) OR Stenosing tenosynovitis[Title/Abstract] Sort by: Publication Date
#3	#1 OR #2
#4	Search (((((((randomized controlled trial[Publication Type]) OR controlled clinical trial[Publication Type]) OR randomized[Title/Abstract]) OR drug therapy[MeSH Subheading]) OR placebo[Title/Abstract]) OR randomly[Title/Abstract]) OR trial[Title/Abstract]) OR groups[Title/Abstract] Sort by: Publication Date
#5	Search (humans[MeSH Terms]) NOT animals[MeSH Terms] Sort by: Publication Date
#6	#4 AND #5
#7	Search“Acupuncture” [Mesh] OR“Moxibustion” Sort by: Publication Date
#8	**Search (((((Acupuncture[Title/Abstract]) OR electroacupuncture[Title/Abstract]) OR auricula acupuncture[Title/Abstract]) OR moxibustion[Title/Abstract]) OR acupuncture and moxibustion[Title/Abstract] Sort by: Publication Date**
#9	#7 OR #8
#10	#3 AND #6 AND #9

#### 3.4.2 Additional search

We will adopt similar retrieval methods to search other sources, such as ongoing or unpublished trials, reference lists of identified publications, and unpublished conference articles.

### 3.5 Data collection

#### 3.5.1 Studies selection

**Figure 1:**
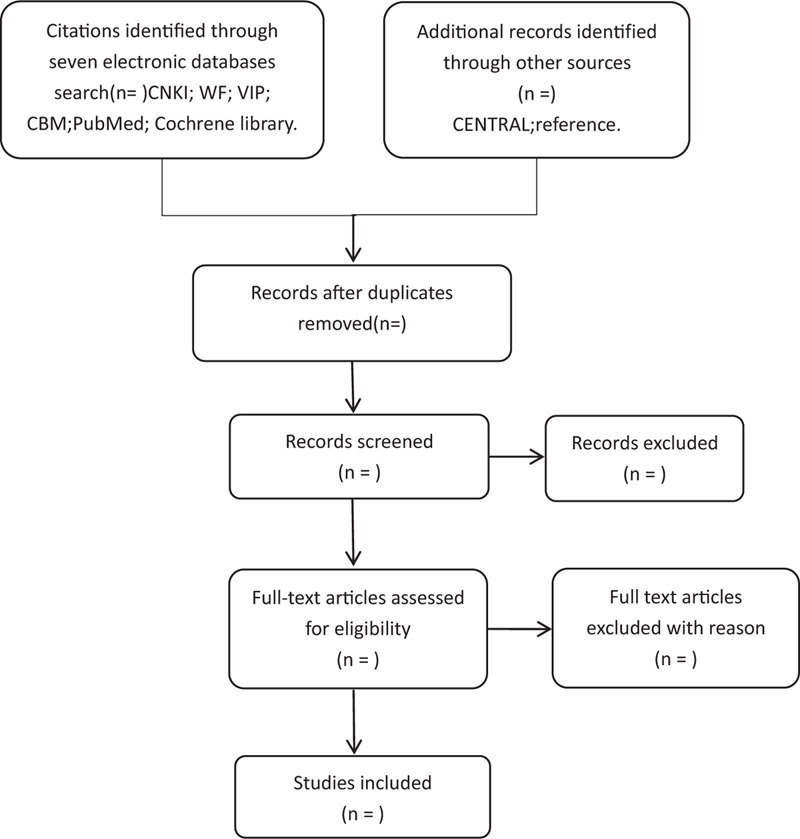
Flowchart of literature selection.

NoteExpress 3.2.0 will be applied to manage the research of electronic searches and other sources. After excluding the duplicated studies, 2 investigators (JX and HFH) will independently review titles and abstracts followed by the selection criteria. Then we will further read the full text for qualified studies if the studies cannot be get rid of in line with the titles and abstracts. For some studies involving unclear information or missing data, QSH will try to contact the authors of original studies for determining whether to include. Finally, 2 investigators will cross-check the selection results. During the procedure, any inconsistency will be resolved through discussion or negotiation to reach for consensus. If no consensus can be reached, we will consult a third investigator (KL). Details for studies selection are shown in a flow chart with Figure 1.

#### 3.5.2 Data extraction

Before data extraction, a standard data extraction form will be designed to by 2 independent investigators (SCL and YHJ), which mainly involves the following information: study design (eg, study type, participants feature, comparison interventions, outcome measures), basic information (eg, title, authors, published time, published journals) and other information in the full text. After completing the extraction, 2 reviewers will cross-check the extraction results. Under the circumstance of some differences, it will be resolved by discussions between the 2 investigators. If still unsolvable, the final judgement will submit to third investigators (KL).

### 3.6 Risk of bias assessment

The quality of the enrolled researches will be assessed by 2 investigators (HFH and JX) respectively adopting the Cochrane Collaboration's tool,^[23]^ which will be evaluated from the following aspects, including generation of random sequences, allocation concealment, application of blind, inadequate outcome data, selective outcome reporting and other possible bias. For the risk of bias in each aspect, it will be rated as high risk, low risk, or unclear risk. After the assessment, 2 investigators will cross-check the rating results strictly. Any discrepancy will be handled via discussion with a third investigator(KL).

### 3.7 Measures of curative effect

We will apply the Review Manager (RevMan 5.3) software to conduct data analysis and quantitative data synthesis. For the results of continuous data, we will measure it using weight mean difference (WMD) with its 95% confidence interval (CI). Meanwhile, we will employ the 95% CI risk ratio (RR) for analyzing the results of dichotomous data.

### 3.8 Management of missing data

When some vital details of methods and results in the included studies missing, the authors will try to contact for the specific information by telephone or email. If the missing data are unable to be obtained, then the analysis will count on those available data, and we will make a narrative analysis of these missing data studies.

### 3.9 Assessment of evidence quality

The GRADE instrumen^[24]^ will be utilized to assess the evidence quality of obtained results, including the primary and secondary results. The quality of specific evidence will be divided into 4 types regarding the level (high, moderate, low or very low) in accordance with 5 parameters (inconsistency, limitations, imprecision, indirectness, and publication bias).^[25]^

### 3.10 Assessment of heterogeneity

The difference between researches in the systematic review is defined as heterogeneity,^[26]^ and the heterogeneity will be assessed by the *I*^2^ test using the RevMan5.3.5 software.^[27,28]^ If the *I*^2^ < 50%, it means slight heterogeneity in these trails. If the *I*^2^≥50%, large heterogeneity will be considered in these trails, and we will attempt to figure out the possible reasons from the aspect of clinic and methodology.

### 3.11 Data analysis

#### 3.11.1 Data synthesis

Before synthesizing the data, the units of each result from different tests will be unified. Then we will conduct statistical analysis for qualified tests using Review Manager V.5.3 statistical software, and the data synthesis and analysis will differ based on the heterogeneity levels. When it came to slight statistical heterogeneity (*I*^2^ < 50%) in the studies, we will use the random-effects model to data synthesis and analysis. If high heterogeneity (*I*^2^ ≥ 50%) in the studies is observed, we will carry out subgroup analysis or sensitivity analysis for identifying the source of heterogeneity. In the meantime, we will perform funnel plots to evaluate the publication bias on the condition that the number of included trials over 10.

#### 3.11.2 Subgroup analysis

If the quantity of included trials is sufficient, subgroup analyses will be conducted to explore the potential sources of heterogeneity, according to the type of AM, the course of treatment, control interventions, as well as the type of De Quervain's tenosynovitis.

#### 3.11.3 Sensitivity analysis

When it comes to significant heterogeneity in our studies, we will perform a sensitivity analysis from the perspective of the character of research, sample size, study design, methodological quality. By continuously excluding some studies, such as low-quality or small sample studies, then we will get a stable result.

#### 3.11.4 Assessment of publication biases

The reporting quality for the included studies will be detected by the 2 review authors using the funnel plots independently. When it comes to asymmetry displayed by the funnel plot, we will apply the Egger method to clarify issues.

## 4 Discussion

De Quervain's tenosynovitis is a work-related musculoskeletal disorder that usually involves pain on the radial side of the wrist, which is exerted by reduplicative and enduring strain of the APL and EPB tendons.^[29]^ The disease often occurs in women with jobs that require repetitive hand movements, such as wringing of cloth, lifting a child. It may also be caused by excessive use of wrists in hobbies or professional work,^[30]^ such as golf player, software engineer, cotton spinner. With the popularity of mobile phones and computers, the incidence rate of De Quervain's tenosynovitis is becoming more common than before. Although the condition of De Quervain's tenosynovitis is not very serious, it will restrict wrist movement and affect the life of people. Modern medical interventions for this disease can be divided into conservative treatments and surgical intervention. Conservative treatments primarily consist of rest, analgesics, therapeutic exercise and splinting.^[31]^ However, the above treatments are often accompanied by corresponding side effects. Thus the substitution and complementary have gradually become patients’ choice.

As an indispensable traditional Chinese therapy, AM for treating diseases of the musculoskeletal system is growing in acceptance by clinicians and is believed to have an analgesic effect.^[32]^ Nevertheless, there are still some limitations in previous studies according to evidence-based medicine, and the effectiveness of AM for Treating De Quervain's tenosynovitis remains controversial. Hence, we plan to perform this systematic review for investigating the efficiency of AM in the treatment of De Quervain's tenosynovitis according to the latest resources. If the results turn out to be valid, it will further help the patients and doctors provide an available option and clinical application.

However, we should acknowledge that there are some limitations to this protocol. Firstly, the types of AM is various in these included studies that may cause high heterogeneity. If possible, we will ensure the consistency of interventions by conducting subgroup analysis. Secondly, many trials are difficult to adopt a blind method when operating the AM, so the risk of bias will be increased to some degree.

Additionally, since the included literature only involves Chinese and English, language bias may also affect the credibility of the results.

## Author contributions

Shouqiang Huang conceived the review protocol and drafted the manuscript. Qian Fan, Jun Xiong revised the study design. Shouqiang Huang, Fanghui Hua, Jie Xiang, Changsheng Li, Hangyu Jin and Kai Liao participated in the design of the search strategy and data extraction data set. Kai Liao, Shouqiang Huang, Qian Fan and Jun Xiong formed the data synthesis and analysis plan. In practice, Kai Liao and Shouqiang Huang will monitor each procedure of the review and are responsible for the quality control. All authors have read and approved the publication of the protocol.

**Conceptualization:** Kai Liao.

**Resources:** Fanghui Hua, Jie Xiang, Changsheng Li, Hangyu Jin.

**Supervision:** qian fan, Jun Xiong.

**Writing – original draft:** Shouqiang Huang.

**Writing – review & editing:** Shouqiang Huang, qian fan, Jun Xiong
